# Endoscopic ultrasound-guided embolization with imatinib for duodenal gastrointestinal stromal tumors in inoperable or treatment-unwilling patients

**DOI:** 10.1055/a-2598-3864

**Published:** 2025-05-28

**Authors:** Beinan Hu, Guilian Cheng, Zhenyun Gong, Duanmin Hu, Longjiang Xu

**Affiliations:** 1 105860 Gastroenterology, Second Affiliated Hospital of Soochow University, Suzhou, China; 2 105860 Pathology, Second Affiliated Hospital of Soochow University, Suzhou, China

Endoscopic ultrasound (EUS)-guided fine-needle aspiration (EUS-FNA) is a promising technique. Although complications are rare, EUS-FNA carries inherent risks, particularly hemorrhage. We report a case of gastrointestinal stromal tumor (GIST) in which EUS-FNA was performed, followed by EUS-guided cyanoacrylate embolization to manage intraprocedural bleeding. Follow-up imaging suggested that this technique may contribute to tumor size reduction.

A 73-year-old man was found to have a hypervascular lesion in the descending portion of the duodenum on computed tomography (CT) ( Fig. 1 ). Gastroscopy revealed the presence of a submucosal tumor, prompting referral to our department for EUS-FNA. EUS revealed a hypoechoic lesion measuring 38.14 × 35.75 mm ( Fig. 2 ). During the second needle puncture, active bleeding started from the tumor. Visualization under gastroscopy was limited, making direct hemostasis difficult. Under EUS and Doppler guidance, 0.5 mL of cyanoacrylate was precisely injected into a vessel within the lesion, resulting in the immediate disappearance of Doppler blood flow signals and successful hemostasis ( Video 1 ). Immunohistochemical analysis confirmed the diagnosis of GIST, with positive staining for CD117 and DOG-1 ( Fig. 3 ).

**Fig. 1:**
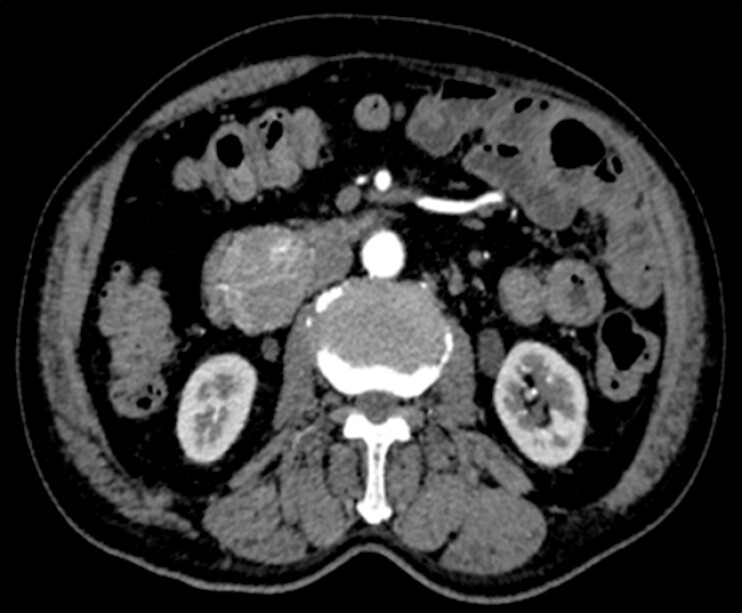
Computed tomography showing a hypervascular lesion in the descending portion of duodenum.

**Fig. 2:**
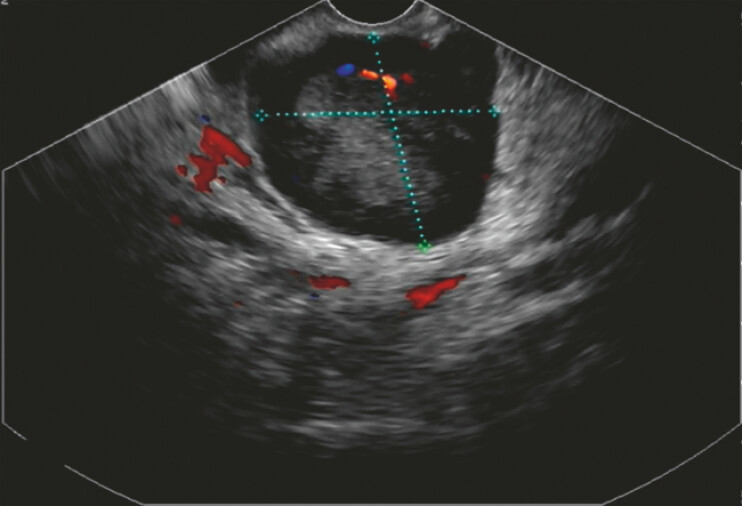
Endoscopic ultrasound revealed a hypoechoic lesion measuring 38.14 × 35.75 mm.

Endoscopic ultrasound (EUS)-guided embolization to control bleeding during EUS-guided fine-needle aspiration (EUS-FNA) of a gastrointestinal stromal tumor (GIST).Video 1

**Fig. 3:**
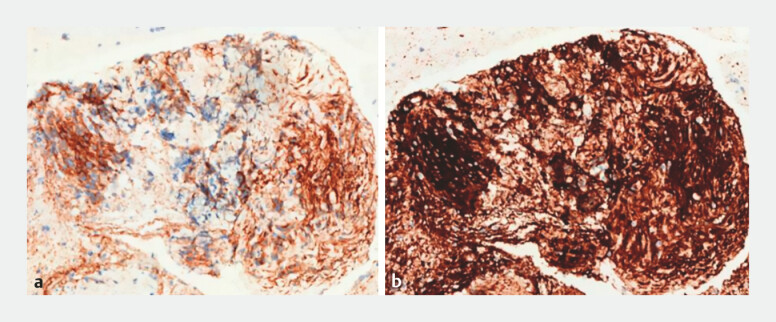
**a–b** Pathological images of the gastrointestinal stromal tumor showing positive staining for CD117 ( **a** ) and DOG-1 ( **b** ).

The patient declined surgical intervention and opted for imatinib therapy at a dose of 400 mg/day. However, due to personal factors, medication adherence was inconsistent. Despite this, no episodes of rebleeding were observed during a 4-month follow-up period. Subsequent gastroscopy and EUS revealed a marked reduction in tumor size ( Fig. 4 ). Follow-up CT demonstrated a decrease in tumor volume and vascularity, along with significant improvement in the compression of the inferior vena cava and adjacent bowel lumen ( Fig. 5 ).

**Fig. 4:**
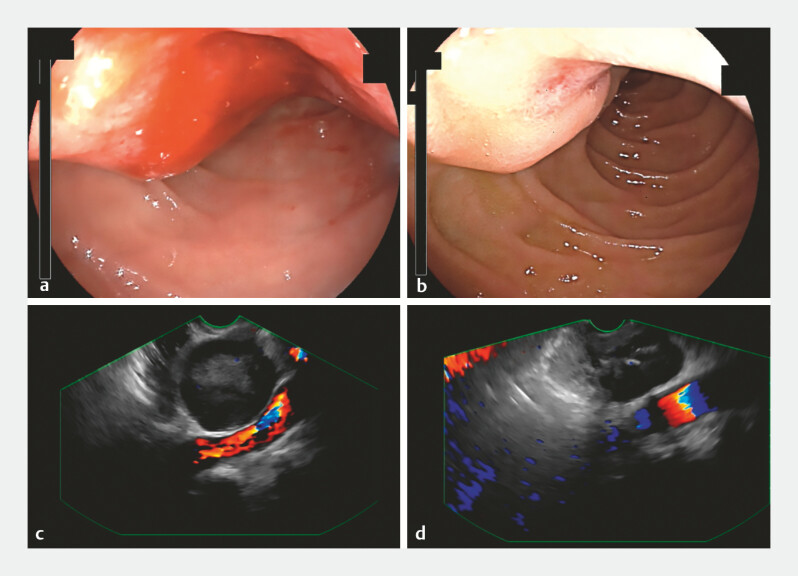
Gastroscopy and endoscopic ultrasound (EUS) images. **a–b** Gastroscopy images performed before treatment ( **a** ) and 4 months after treatment ( **b** ). **c–d** EUS images performed before treatment ( **c** ) and 4 months after treatment ( **d** ).

**Fig. 5:**
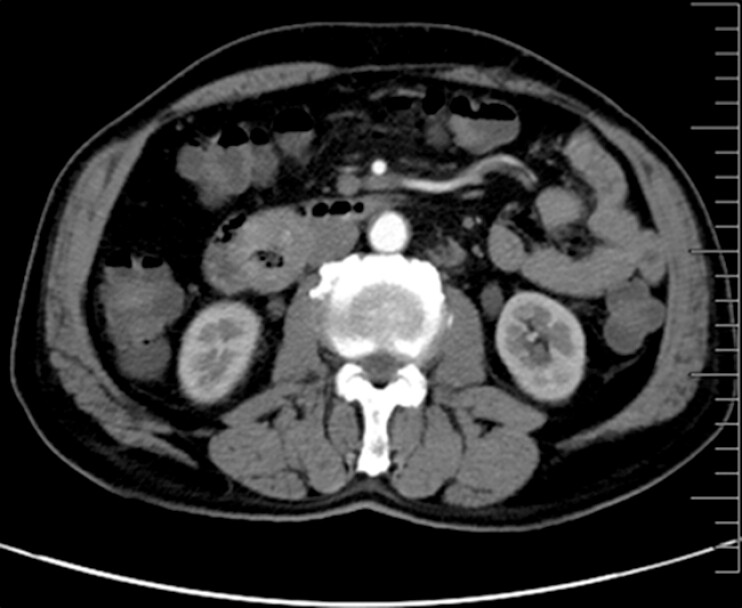
Computed tomography performed 4 months after treatment.

The overall complication rate of EUS-FNA ranges from 0–2.5%, with hemorrhage occurring in approximately ≤2% of cases 1 . Standard hemostatic approaches include hemoclips and electrocautery. In our case, EUS-guided cyanoacrylate embolization achieved effective hemostasis. We propose that EUS-guided embolization may offer the additional benefit of controlling bleeding. Despite the patientʼs inconsistent medication adherence, the tumor size decreased significantly. Therefore, we suggest that EUS-guided embolization can lead to tumor size reduction by occluding the tumor blood supply. However, due to the rarity of this condition, we have not conducted further studies. Further clinical studies are needed to determine the efficacy of EUS-guided embolization.

Endoscopy_UCTN_Code_CPL_1AL_2AB

## Notes

Endoscopy E-Videos https://eref.thieme.de/e-videos
*E-Videos* is an open access online section of the journal *Endoscopy* , reporting on interesting cases and new techniques in gastroenterological endoscopy. All papers include a high-quality video and are published with a Creative Commons CC-BY license. Endoscopy E-Videos qualify for HINARI discounts and waivers and eligibility is automatically checked during the submission process. We grant 100% waivers to articles whose corresponding authors are based in Group A countries and 50% waivers to those who are based in Group B countries as classified by Research4Life (see: https://www.research4life.org/access/eligibility/ ). This section has its own submission website at https://mc.manuscriptcentral.com/e-videos .
